# Supplementary material for: Rapid expansion of Treg cells protects from collateral colitis following a viral trigger
Source: Nat Commun. 2020 Mar 23;11:1522. doi: 10.1038/s41467-020-15309-6 (PMC7090079; doi:10.1038/s41467-020-15309-6)
Supplement: Supplementary file 1 — Supplementary Information [file 41467_2020_15309_MOESM1_ESM.pdf]

SUPPLEMENTARY INFORMATION

Rapid expansion of Treg cells protects from collateral colitis following a viral trigger

Schorer et al.

* Corresponding author: nicole.joller@immunology.uzh.ch

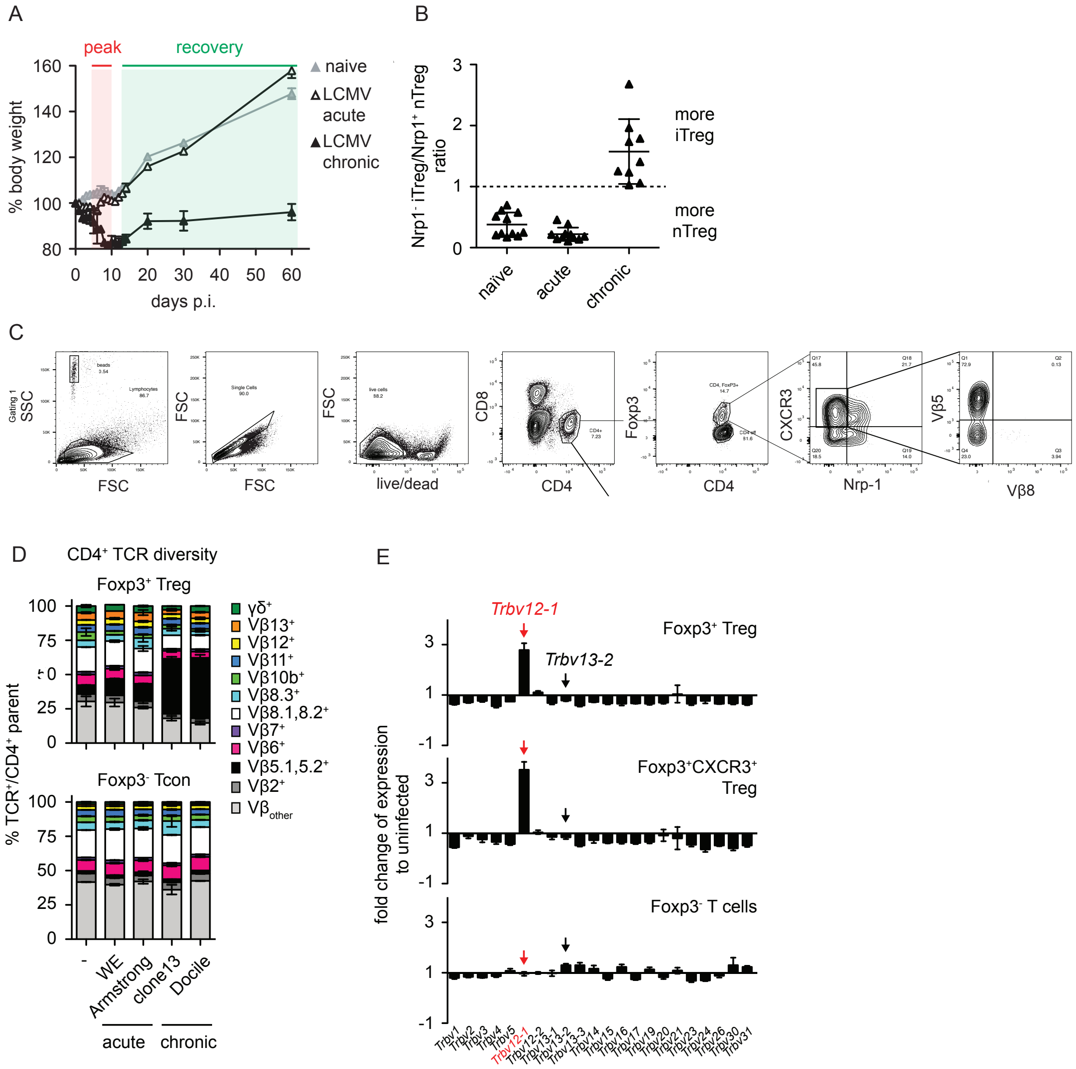

Supplementary Figure 1. TCR usage of Treg cells during LCMV infection. (A-B) WT mice were infected with 200 f.f.u. LCMV WE (acute), 10^6 f.f.u. LCMV clone 13 (chronic), or were left naïve. (A) Body weight was monitored throughout the course of infection and (B) the ratio of $CD4^+Foxp3^+CXCR3^+Nrp1^-$ iTreg cells to $CD4^+Foxp3^+CXCR3^+Nrp1^+$ nTreg cells was determined by flow cytometry on day 14. (C) Gating strategy for the different Treg populations. (D) WT mice were infected with LCMV WE (200 f.f.u.), Armstrong (10^6 f.f.u.), clone 13 (10^6 f.f.u.), or Docile (10^6 f.f.u.) or were left naïve and frequencies of T cells expressing the indicated Vβ chains among $CD4^+Foxp3^+$ Treg or $CD4^+Foxp3^-$ conventional T cells were determined by flow cytometry on day 10 post infection. (E) Transcriptional levels of *Trbv* gene segments in $CD4^+Foxp3^+$ Treg, $CD4^+Foxp3^+CXCR3^+$ Treg or $CD4^+Foxp3^-$ conventional T cells isolated from naïve or LCMV infected (200 f.f.u. WE) mice were determined by RNA sequencing and are displayed as fold change to naïve.

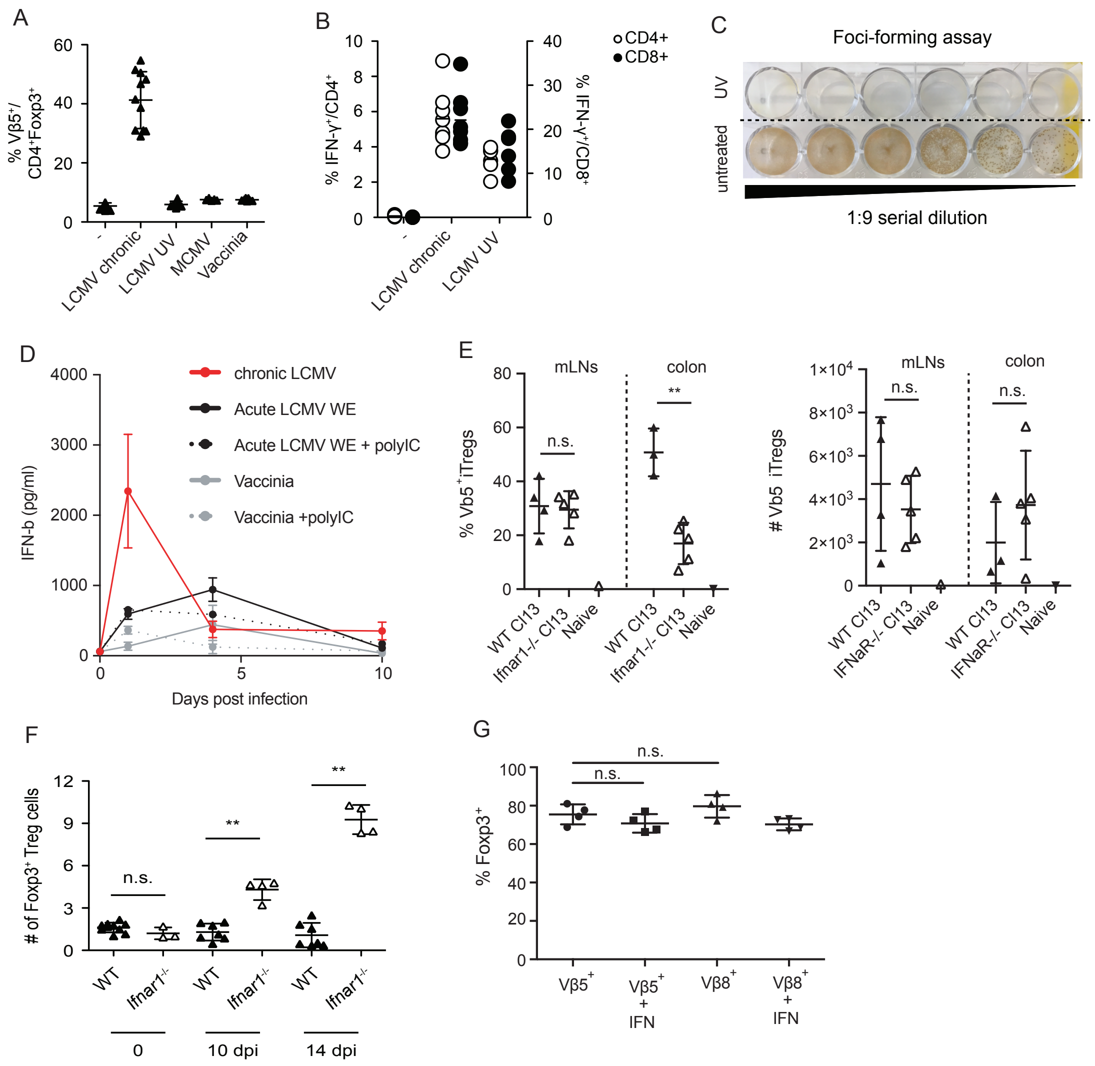

Supplementary Figure 2. Effect of type I IFN on Treg composition. (A-B) WT mice were either infected acutely with Vaccinia virus, chronically with LCMV clone 13 or MCMV, or immunized with a high dose of UV-inactivated LCMV clone 13, or were left naïve. On day 14, TCR Vβ5$^+$ frequencies among CD4$^+$Foxp3$^+$ Treg cells were determined by flow cytometry (A). In LCMV infected and UV-LCMV immunized mice the IFN-γ production in splenocytes upon LCMV-specific re-stimulated (gp61+gp33 peptides) was also determined in CD4$^+$ (open symbols) or CD8$^+$ (filled symbols) T cells (B). (C) Foci-forming assay of unmanipulated or UV-inactivated LCMV inoculum. (D) WT mice were infected with Vaccinia virus (10^6 f.f.u.), Vaccinia Virus + poly IC (2 x 50μg/mouse, day 0 and 2), LCMV WE (200 f.f.u.), LCMV WE + poly IC, or LCMV clone 13 (10^6 f.f.u.) and type I IFN levels in the blood were determined over time. (E-F) WT or *Ifnar1*$^{-/-}$ mice were infected with LCMV clone 13 (10^6 f.f.u.) and frequencies and total numbers of Vβ5$^+$CD4$^+$Foxp3$^+$CXCR3$^+$Nrp1$^-$ iTreg cells in mesenteric LNs and colon (E) as well as total numbers of splenic CD4$^+$Foxp3$^+$ Treg cells (F) were determined by flow cytometry. (G) Sorted CD4$^+$Foxp3$^-$ T cells from naïve *Foxp3*-GFP.KI mice were polarized towards iTregs with plate bound anti-CD3/anti-CD28 and TGF-β in the presence or absence of type I IFN. On day 2 IL-2 was added and induction of Foxp3 in Vβ5$^+$ or Vβ8$^+$ CD4$^+$ T cells was determined by flow cytometry on day 5. Data are presented as mean±SD.

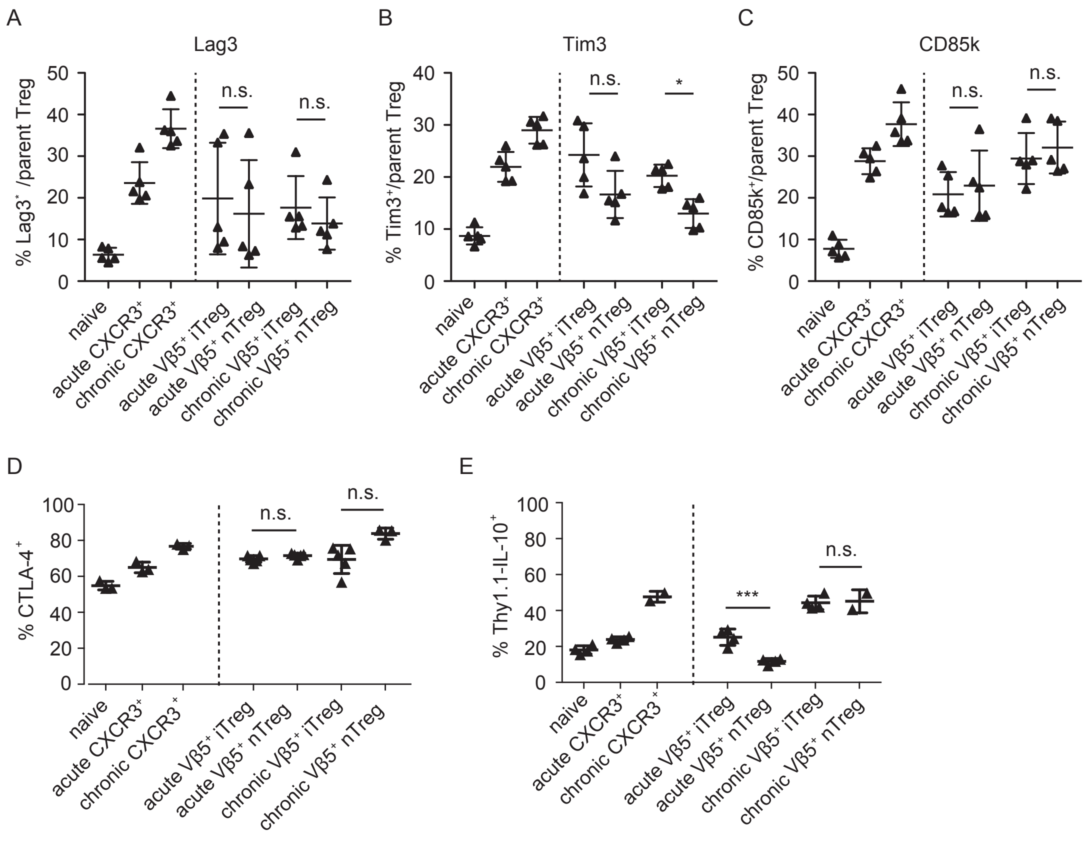

Supplementary Figure 3. Expression of Treg signature molecules by Treg subsets. *Foxp3*-GFP.KI x IL-10-Thy1.1 reporter mice were infected with LCMV WE (200 f.f.u., acute) or clone 13 (10^6 f.f.u., chronic) or were left naïve. Frequencies of (A) Lag-3$^+$, (B) Tim3$^+$, (C) CD85k$^+$, (D) CTLA-4$^+$ and (E) IL-10-Thy1.1$^+$ cell among the indicated CD4$^+$Foxp3$^+$ Treg subsets were determined by flow cytometry. Summary data are shown as mean±SD, n=5. For statistics, Mann-Whitney U was used.

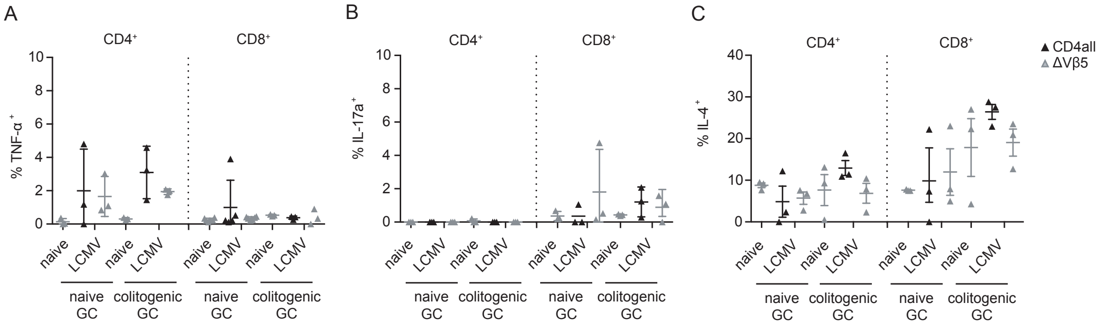

Supplementary Figure 4. T cell response in mice with autophagy-deficient DCs. *Atg5*$^{fl/fl}$x*Itgax*$^{Cre/-}$ (cKO) as well as *Atg5*$^{fl/WT}$x*Itgax*$^{Cre/-}$ (het) and *Atg5*$^{fl/f}$ (WT) controls were infected with LCMV WE (200 f.f.u., acute) or clone 13 (10^6 f.f.u., chronic) or were left naïve. (A) Total counts of splenic CD4$^+$ and CD8$^+$ T cells were determined by flow cytometry. (B) Viral titers in indicated organs were determined on day 14 by foci-forming assay. (C-E) Where indicated, mice were additionally continuously treated with antibiotics staring 2 weeks before infection. On day 14, total counts of splenic CD4$^+$Foxp3$^+$ Treg cells (C) as well as frequencies of Vβ5$^+$CD4$^+$Foxp3$^+$CXCR3$^+$Nrp1$^-$ (D) and Vβ8$^+$CD4$^+$Foxp3$^+$CXCR3$^+$Nrp1$^-$ (E) iTreg cells were determined by flow cytometry. Data are shown as mean±SD; summary graphs display data of 2-3 independent experiments. For statistics, Mann-Whitney U was used.

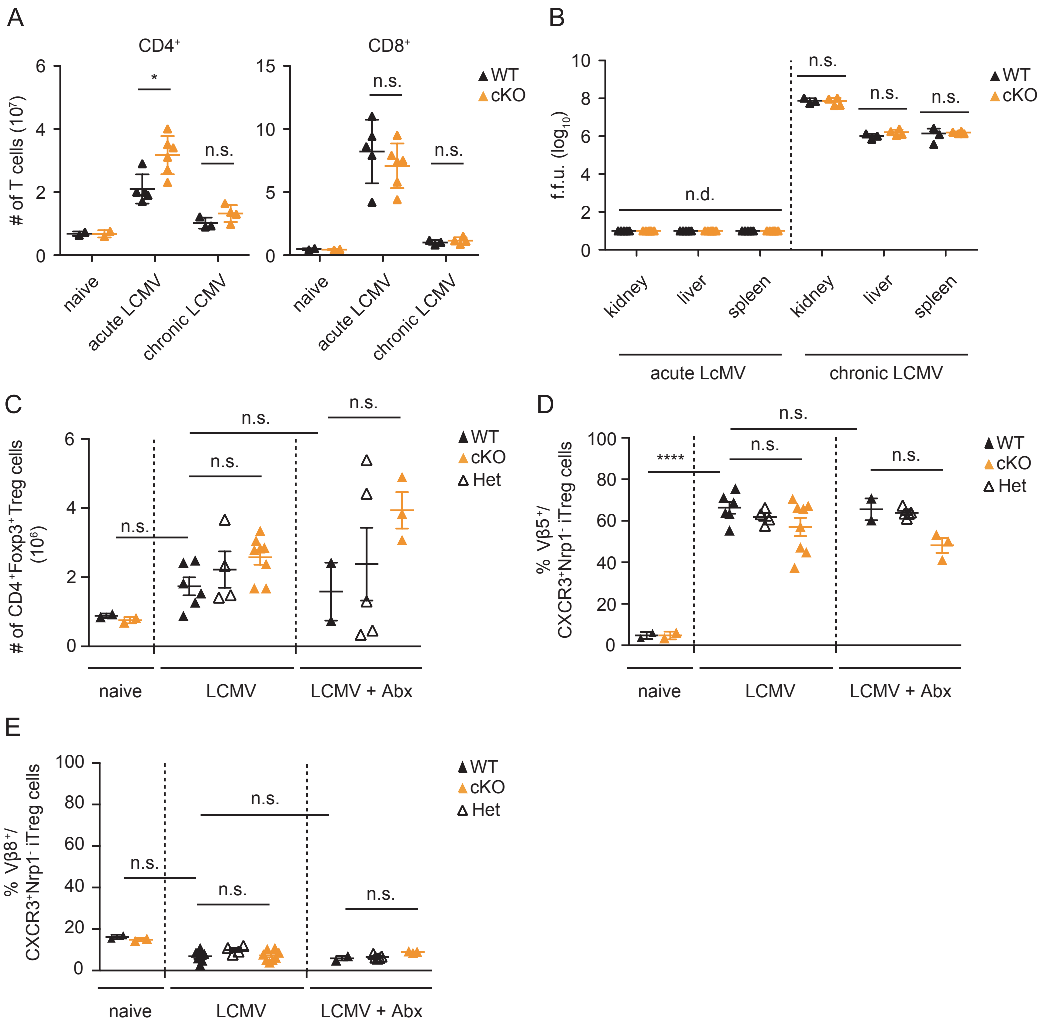

Supplementary Figure 5. Treg cell loss early during infection. (A) *Tcrb*$^{-/-}$*Tcrd*$^{-/-}$ mice were reconstituted with wild-type CD8^{+} T cells together with either total CD4^{+} T cells or Vβ5^{-}CD4^{+} T cells, infected with LCMV clone 13 (10^{6} f.f.u.) or left naïve, and analyzed 10 or 17 days after infection. Expression of Foxp3 and TCR Vβ5.1,5.2 among CD4^{+} T cells was determined by flow cytometry. (B) *Nr4a1*-GFP mice were infected with LCMV WE (200 f.f.u.) and analyzed for *Nr4a1*-GFP^{+} and TCR Vβ5.1,5.2 expression in CD4^{+}Foxp3^{+}CXCR3^{+}Nrp1^{-} iTreg (left) and CD4^{+}Foxp3^{-} conventional T cells (right) by flow cytometry.

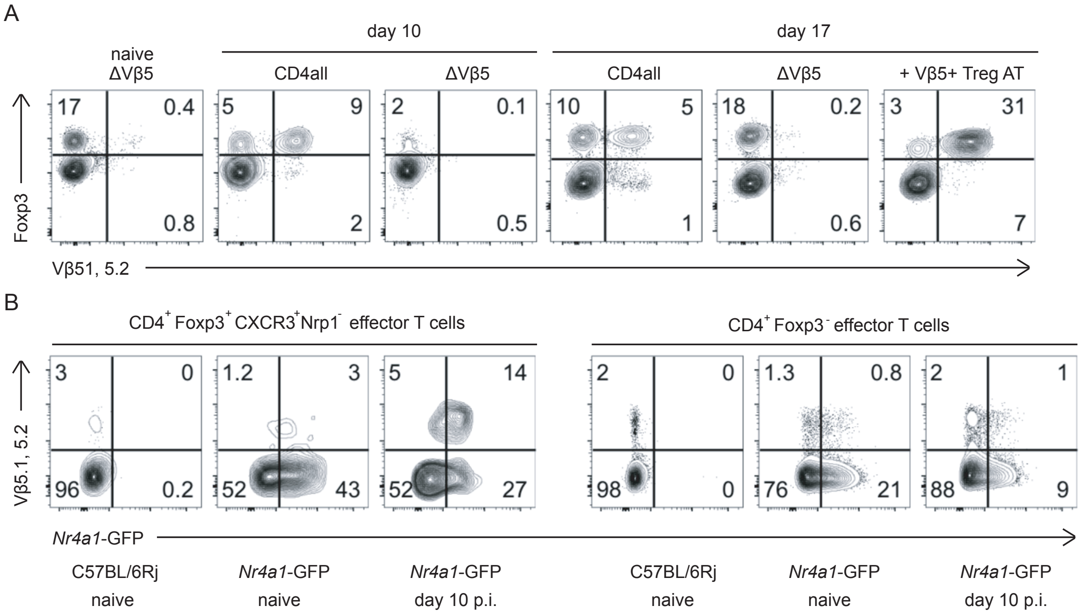

Supplementary Figure 6. T cells response in colitic mice. *Tcrb*$^{-/-}$*Tcrd*$^{-/-}$ mice reconstituted with total CD4^{+} T cells (CD4 all) or Vβ5^{-} CD4^{+} T cells (ΔVβ5) were left naïve infected with LCMV (10^{6} f.f.u. clone 13) and on day 10 splenocytes were re-stimulated with gut content for 6 hrs. Brefeldin A was added for the last 4 h and frequencies of (A) TNF-α^{+}, (B) IL-17^{+}, and (C) IL-4^{+} CD4^{+} and CD8^{+} T cells were determined by flow cytometry. Data are shown as mean±SD.

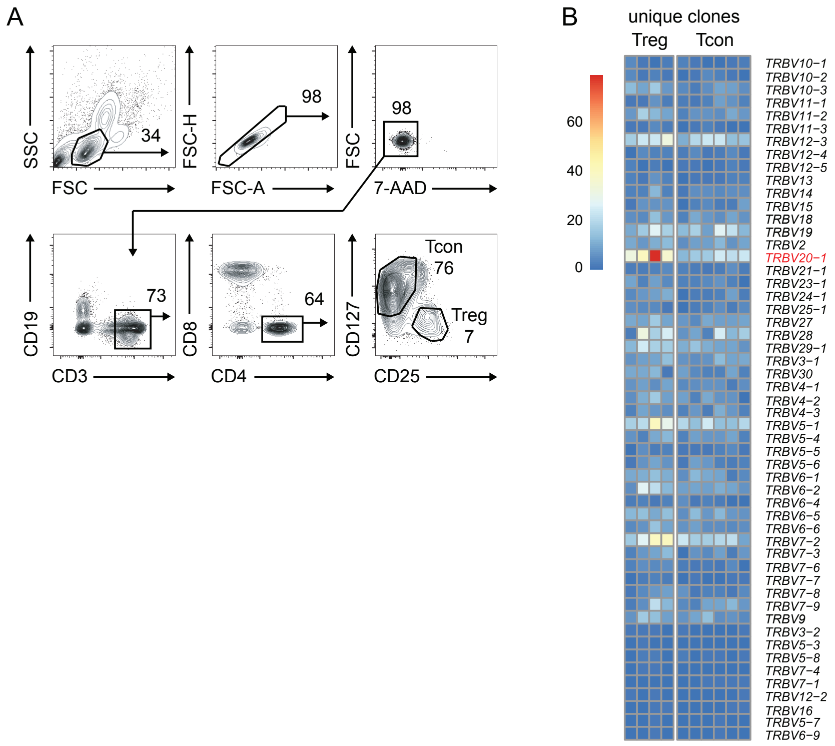

Supplementary Figure 7. TCR repertoire of human T cells. T cells from PBMCs of 6 healthy donors were flow sorted and analyzed by RNA-Sequencing. (A) Gating strategy of CD4^{+}CD127^{-}CD25^{hi} Treg cells and CD4^{+}CD127^{var}CD25^{var} conventional T cells. (B) The number of unique TCR clones within all Vβ segments was determined based on CDR3 sequences extracted from RNA sequencing data. Scale bars refer to the number of unique clones.
