# Supplementary material for: Rapid expansion of Treg cells protects from collateral colitis following a viral trigger
Source: Nat Commun. 2020 Mar 23;11:1522. doi: 10.1038/s41467-020-15309-6 (PMC7090079; doi:10.1038/s41467-020-15309-6)
Supplement: Supplementary file 3 — Reporting Summary [file 41467_2020_15309_MOESM3_ESM.pdf]

nature research

Corresponding author(s): Nicole Joller

Last updated by author(s): Feb 17, 2020

Reporting Summary

Nature Research wishes to improve the reproducibility of the work that we publish. This form provides structure for consistency and transparency in reporting. For further information on Nature Research policies, see Authors & Referees and the Editorial Policy Checklist.

Statistics

For all statistical analyses, confirm that the following items are present in the figure legend, table legend, main text, or Methods section.

n/a	Confirmed	
☐	☒	The exact sample size (*n*) for each experimental group/condition, given as a discrete number and unit of measurement
☐	☒	A statement on whether measurements were taken from distinct samples or whether the same sample was measured repeatedly
☐	☒	The statistical test(s) used AND whether they are one- or two-sided<br>*Only common tests should be described solely by name; describe more complex techniques in the Methods section.*
☒	☐	A description of all covariates tested
☐	☒	A description of any assumptions or corrections, such as tests of normality and adjustment for multiple comparisons
☐	☒	A full description of the statistical parameters including central tendency (e.g. means) or other basic estimates (e.g. regression coefficient) AND variation (e.g. standard deviation) or associated estimates of uncertainty (e.g. confidence intervals)
☐	☒	For null hypothesis testing, the test statistic (e.g. *F*, *t*, *r*) with confidence intervals, effect sizes, degrees of freedom and *P* value noted<br>*Give P values as exact values whenever suitable.*
☒	☐	For Bayesian analysis, information on the choice of priors and Markov chain Monte Carlo settings
☐	☒	For hierarchical and complex designs, identification of the appropriate level for tests and full reporting of outcomes
☐	☒	Estimates of effect sizes (e.g. Cohen's *d*, Pearson's *r*), indicating how they were calculated

Our web collection on statistics for biologists contains articles on many of the points above.

Software and code

Policy information about availability of computer code

Data collection	FACS Diva
Data analysis	GraphPad Prism 6, Subread (v1.6.2), edgeR, MiXCR (version 3.0.3), FlowJo 10

For manuscripts utilizing custom algorithms or software that are central to the research but not yet described in published literature, software must be made available to editors/reviewers. We strongly encourage code deposition in a community repository (e.g. GitHub). See the Nature Research guidelines for submitting code & software for further information.

Data

Policy information about availability of data

All manuscripts must include a data availability statement. This statement should provide the following information, where applicable:

- Accession codes, unique identifiers, or web links for publicly available datasets
- A list of figures that have associated raw data
- A description of any restrictions on data availability

Source data for all figures and supplementary figures are provided with the paper. RNA-Seq data that support the findings of this study have been deposited at the ArrayExpress database at EMBL-EBI (www.ebi.ac.uk/arrayexpress) and are available via the accession numbers E-MTAB-6156 (mouse Tregs) and E-MTAB-8819 (human Tregs).

Field-specific reporting

Please select the one below that is the best fit for your research. If you are not sure, read the appropriate sections before making your selection.

☒ Life sciences ☐ Behavioural & social sciences ☐ Ecological, evolutionary & environmental sciences

For a reference copy of the document with all sections, see nature.com/documents/nr-reporting-summary-flat.pdf

Life sciences study design

All studies must disclose on these points even when the disclosure is negative.

Sample size	We assumed an average effect size of 30-50%, and a difference of 10-15% as biologically significant. The desired power was 90%, the significance level was set to p<0.05%, the variance was 2.5 for systemic infections (based on experience). Experimental groups were compared to control groups so a two sided test is used. Using the Pwr package in R this gave us a group size of 2.1-2.7 and we thus generally used groups of at least 3 animals for systemic infections. As different transgenic and KO animals were used in this study, in certain cases, the group size was decreased to two or increased, depending on the number of animals with the desired genotype.
Data exclusions	No data was excluded from the analyses. Individual biological experiments or animals were not included in the analyses when both the cytokine data and pathology data indicated that animals were not infected.
Replication	All biological experiments were performed at least twice, for most figures at least one experimental repeat was performed by a different person. All attempts at data replication were successful.
Randomization	Experimental groups were not randomized, but age-matched and housed in the same animal house for the duration of the experiment. HUman donors were age and sex matched between groups.
Blinding	All histo-pathological data was single-blinded for the analysis. For other experiments, groups were not blinded as treatment of the animals had to be noted on the cage cards due to legal restrictions.

Reporting for specific materials, systems and methods

We require information from authors about some types of materials, experimental systems and methods used in many studies. Here, indicate whether each material, system or method listed is relevant to your study. If you are not sure if a list item applies to your research, read the appropriate section before selecting a response.

Materials & experimental systems

n/a	Involved in the study	
☐	☒	Antibodies
☒	☐	Eukaryotic cell lines
☒	☐	Palaeontology
☐	☒	Animals and other organisms
☐	☒	Human research participants
☒	☐	Clinical data

Methods

n/a	Involved in the study	
☒	☐	ChIP-seq
☐	☒	Flow cytometry
☒	☐	MRI-based neuroimaging

Antibodies

Antibodies used	All fluorescently labeled antibodies - for murine samples: CD4 (RM4-5 or GK1.5), CD8 (53-6.7), CXCR3 (CXCR3-173), Nrp1 (3E12), Foxp3 (FJK-16s), TCR Vβ5.1,5.2 (MR9-4), Vβ8.1,8.2 (KJ16-133.18), TCR γδ (GL3/UC7-13D5), Vβ2 (B20.6), Vβ6 (RR4-7), Vβ7 (TR310), Vβ8.3 (1B3.3), Vβ10b (B21,5), Vβ11 (RR3-15/KT11), Vβ12 (MR11-1), and Vβ13 (MR12-4), CD44 (IM7), CD62L (MEL-14), TIGIT (1G9), LAG3 (C9B7W), PD-1 (J43), CD39 (Duha59), CD73 (TY/11.8), CD85k (H1.1), BrdU (Bu20a), IFN-γ (XMG1.2), CD5 (53-7.3), CD11c (N418), MHCII (M5/114.15.2), CD45.1 (A20), CD90.1 (OX-7); for human samples: CD3 (OKT3), CD4 (SK3), CD8 (SK1), CD19 (HIB19), CD25 (M-A251), CD45RA (HI100), and CD127 (A019D5) - were purchased from Biolegend, eBioscience, or BD Biosciences. For T cell spectratyping from human PBMCs, the IOTest Beta Mark TCR Vβ Repertoire Kit was used (Beckman Coulter). To detect live cells, the Zombie-NIR fixable dye (for murine cells) and 7-AAD (for human cells) were used.
Validation	All antibodies used are standard antibodies and have been validated by the manufacturers. They were titrated in house.

Animals and other organisms

Policy information about studies involving animals; ARRIVE guidelines recommended for reporting animal research

Laboratory animals	C57BL/6Rj, Foxp3-GFP.KI reporter mice, B6.Cg-Tg(TcraTcrb)425Cbn/J (OT-II; JAX 00419454) mice, B6.Cg-Gpi1aThy1aIgha/J mice (Thy1.1; JAX 00131755), Ifnar1tm1Agt mice (MMRC JAX 3204556), Tcrbtm1MomTcrdtm1Mom mice (JAX 00212257), and

	C57BL/6-Tg(Nr4a1-EGFP/cre)820Khog/J mice (JAX 01661742) . B6.SJL-PtprcaPepcb/BoyJ (Ly5.1; JAX 002014) and Foxp3-GFP.KI mice were crossed to generate Foxp3-GFP.KILy5.1 reporter mice. Atg5flox/flox and C57BL/6J-Tg(Itgax-cre,-EGFP)4097Ach/J mice (JAX 00756759) were crossed to obtain Atg5fl/flxItgaxCre/- mice<br>Mice were age and gender matched within experiments and used at 8-16 weeks of age.
Wild animals	none
Field-collected samples	none
Ethics oversight	the cantonal veterinary office of Zurich (permit number ZH100/2014, ZH168/2015, ZH114/2017, ZH119/2017and ZH179/2017)

Note that full information on the approval of the study protocol must also be provided in the manuscript.

Human research participants

Policy information about studies involving human research participants

Population characteristics	for samples used for sequencing, samples were recruited from gender and age matched healthy volunteers<br>samples from colitis patients were collected from patients with active and inactive disease and groups were matched for gender and age, the healthy controls for these samples were matched for gender and age to the patient samples
Recruitment	Samples for healthy donors were recruited from volunteers, patients were recruited through the Swiss IBD Cohort
Ethics oversight	Cantonal Ethics Committee of Zurich, BASEC number 2016-01440 and EK-1316

Note that full information on the approval of the study protocol must also be provided in the manuscript.

Flow Cytometry

Plots

Confirm that:

☒ The axis labels state the marker and fluorochrome used (e.g. CD4-FITC).

☒ The axis scales are clearly visible. Include numbers along axes only for bottom left plot of group (a 'group' is an analysis of identical markers).

☒ All plots are contour plots with outliers or pseudocolor plots.

☒ A numerical value for number of cells or percentage (with statistics) is provided.

Methodology

Sample preparation	Single cell suspensions were obtained by physical disruption (spleen, LN) or digestion with collagenase/DNAse (colon) and cells were used directly for extracellular staining (45 mins at 4°C) or restimulated ex vivo. For transcription factor staining the Foxp3 Staining Buffer Set (Thermo Fisher) and for intracellular cytokine staining the BD Fixation/Permeabilization Solution kits (BD Bioscience) were used according to the manufacturer's instructions.
Instrument	LSRFortessa or FACSCanto II cytometers were used for cell acquisition and a FACS Aria III 5L was used for cell sorting (all BD Bioscience)
Software	Data were acquired with Diva Software (BD Bioscience) and analyzed with FolwJo (TreeStar).
Cell population abundance	All sorted populations reached a purity of >99%. Purity was controlled by recording a sample of the sorted population post sort.
Gating strategy	Gates were set based on FMO or isotype stained samples. The most relevant gating strategies are depicted in the supplementary figures

☒ Tick this box to confirm that a figure exemplifying the gating strategy is provided in the Supplementary Information.
